# Genetic Counseling for Childhood Cancer Predisposition Syndromes: A Six-Year Retrospective Study

**DOI:** 10.3390/children13060793

**Published:** 2026-06-09

**Authors:** Milena Stoyanova, Dinnar Yahya, Mari Hachmeriyan, Mariya Levkova

**Affiliations:** 1 Department of Medical Genetics, Medical University Varna, Marin Drinov Str 55, 9000 Varna, Bulgaria; 2 Laboratory of Medical Genetics, University Multiprofile Hospital for Active Treatment “St. Marina”, Hristo Smirnenski Blv 1, 9000 Varna, Bulgaria

**Keywords:** cancer predisposition syndromes, pediatric oncology, genetic counseling, RASopathies

## Abstract

**Background**: Cancer predisposition syndromes (CPS) are increasingly recognized in pediatric oncology. In many cases, malignancy in children represents one manifestation of a broader genetic syndrome, often accompanied by dysmorphic features or other distinctive clinical findings. **Methods**: Clinical documentation and genetic test reports of pediatric patients evaluated over a six-year period (2020–2025) at a genetic counseling unit in a tertiary university hospital in Varna, Bulgaria, were retrospectively reviewed. Referral patterns, clinical characteristics, and genetic findings in children assessed for suspected CPS were analyzed. **Results**: In total, 430 children underwent genetic testing during the study period; among them, 42 fulfilled the criteria for CPS and were subsequently included in the analysis. Patients were categorized into three groups: those with malignancy (21.4%), those with high-risk hematologic/immune features without malignancy (9.5%), and those referred based on phenotypic features alone (69.0%). Most referrals originated outside oncology services, primarily from general pediatric clinics and outpatient settings, highlighting the importance of non-oncologists in early CPS recognition. Multisystem phenotypic features were common, with 69.0% of patients exhibiting involvement of two or more clinical domains. Genetic testing, predominantly using multigene panels and exome sequencing, identified clinically relevant variants in established CPS genes, most frequently in autosomal dominant conditions within this diagnosis-confirmed cohort. The most common diagnostic categories included NF1 spectrum disorders and RASopathies. **Conclusions**: These findings emphasize that CPS identification often relies on recognition of non-oncologic features rather than malignancy alone. Genetic counseling plays a central role in diagnosis, risk assessment, and cascade testing. Strengthening awareness among general pediatricians and improving access to genetic services are critical for optimizing early detection and prevention strategies.

## 1. Introduction

Cancer remains the leading cause of disease-related mortality in children and adolescents after the neonatal stage [1]. Increasing evidence indicates that cancer predisposition syndromes (CPS) play a significant role in pediatric oncology, with an estimated 8–13% of children with cancer harboring an underlying genetic susceptibility [2]. These syndromes arise from germline pathogenic variants that confer an increased lifetime risk of malignancy, distinguishing them from sporadic cancers, which typically result from acquired somatic mutations [3]. Importantly, hereditary cancers are not always inherited; a substantial proportion may result from de novo germline mutations, which limits the utility of family history alone in identifying at-risk individuals [4].

In children who develop cancer, suspicion for an underlying predisposition may be raised by clinical features such as bilateral or multifocal primary tumors, unusually early age at diagnosis, or the occurrence of rare tumor types, including pheochromocytoma, adrenocortical carcinoma, tumors of plexus choroideus, rhabdoid tumors and retinoblastoma [3].

Importantly, malignancy in children may also represent one component of a broader genetic syndrome characterized by dysmorphic features or distinctive physical findings. For example, children with Noonan syndrome may present with characteristic facial features, congenital heart defects, and an increased risk of certain malignancies [5], while the presence of café-au-lait macules is a hallmark feature of Neurofibromatosis type 1, a condition associated with an elevated risk of both benign and malignant tumors [5]. Recognition of such phenotypic features is essential for early identification of underlying cancer predisposition. Moreover, due to the increasing implementation of genetic diagnostics in general pediatric care for a wide range of conditions, children may be incidentally identified as being at increased cancer risk due to the detection of pathogenic or likely pathogenic germline variants [6].

In some cases, specific tumor subtypes are closely linked to particular hereditary syndromes and are an indication for genetic counseling. For example, adrenal carcinoma is characteristic of Li–Fraumeni syndrome in pediatric cases [7], while the different subtypes of medulloblastoma are commonly associated even with specific variants in specific genes [8]. However, not all pediatric tumors are syndrome-specific. For example, Wilms tumor (nephroblastoma), one of the most common childhood malignancies, typically occurs sporadically but may also be a manifestation of various hereditary conditions [9]. Syndromic forms of Wilms tumor are observed in disorders such as Beckwith–Wiedemann syndrome, WAGR syndrome, Denis–Drash syndrome, and Fanconi anemia [9].

Advances in high-throughput next-generation sequencing (NGS) technologies have significantly improved the detection of germline alterations in pediatric cancer patients [10]. Current data indicate that approximately 8.5% of pediatric tumors are associated with identifiable germline mutations [10]. This growing recognition of inherited susceptibility underscores the critical importance of integrating genetic evaluation into pediatric oncology care. In this context, genetic counseling plays a central role in risk assessment, interpretation of genetic findings, guidance on surveillance strategies, and support for affected children and their families [4].

This study aims to present the six-year experience of the genetic counseling unit at a University Hospital in Varna, Bulgaria, and to illustrate its clinical workflow, including patient referral patterns, indications for genetic testing, and the integration of genetic counseling into pediatric oncology care.

## 2. Materials and Methods

### 2.1. Study Design and Setting

This retrospective observational study was conducted at the Laboratory of Medical Genetics and the genetic counseling unit at the University Hospital “St. Marina”, Varna, Bulgaria. Clinical documentation and genetic test reports of pediatric patients evaluated between 1 January 2020, and 31 December 2025, were reviewed. The study was performed in accordance with the Declaration of Helsinki and local institutional policies. Genetic testing was conducted following written informed consent from parents/legal guardians (and assent when applicable).

Cancer predisposition syndromes were defined as genetic conditions associated with an increased risk of developing malignancy based on established literature and current clinical guidelines. Conditions were classified as CPS if they met at least one of the following criteria:

(i) well-established association with increased cancer risk in published literature;
(ii) inclusion in recognized CPS gene lists or surveillance guidelines; or
(iii) documented association between the identified gene and tumor development in clinical databases or peer-reviewed studies.

Examples include, but are not limited to, neurofibromatosis type 1, RASopathies (e.g., Noonan syndrome), Li–Fraumeni syndrome, Beckwith–Wiedemann syndrome, and DNA repair disorders.

### 2.2. Participants (Eligibility Criteria)

Children and adolescents (≤18 years) evaluated in our genetic counseling unit during the study period were eligible for the source population if they received a confirmed genetic diagnosis following genetic testing performed as part of routine care. Referrals to our unit covered a broad spectrum of pediatric indications and were not restricted to suspected cancer predisposition. From this source population, we defined the analytic CPS cohort as those patients whose confirmed molecular diagnosis met predefined criteria for a cancer/tumor predisposition syndrome (CPS). Patients were included regardless of whether a malignant tumor was present at referral. Cases with incomplete clinical documentation and/or unavailable genetic test reports and those with constitutional aneuploidies (e.g., trisomy 21) were excluded. Although referrals were not limited to CPS suspicion, common referral triggers among CPS-diagnosed patients included: (i) personal history of malignancy, particularly early-onset, rare, bilateral, or multifocal tumors; (ii) clinical features suggestive of CPS, including pigmentary abnormalities (e.g., café-au-lait macules), dysmorphic features, congenital anomalies, growth disturbances (overgrowth or short stature), and neurodevelopmental delay; (iii) suspicion of a specific hereditary syndrome associated with increased cancer risk (e.g., neurofibromatosis type 1, Noonan syndrome, Beckwith–Wiedemann spectrum, Li–Fraumeni syndrome); (iv) hematologic or immune abnormalities raising concern for inherited predisposition, including persistent cytopenias, suspected bone marrow failure syndromes, or immune dysregulation; or (v) cascade testing in first-degree relatives of individuals with a known cancer predisposition diagnosis.

### 2.3. Genetic Testing and Variant Interpretation

Genetic testing was performed in accredited European reference laboratories operating under ISO certification standards using approaches selected according to clinical indication, including whole exome sequencing (WES)/clinical exome sequencing, targeted gene panels, and imprinting/methylation analyses. Variants were interpreted according to standard clinical frameworks (e.g., ACMG/AMP [11]) and classified as pathogenic, likely pathogenic, or variants of uncertain significance (VUS). For analysis, variants were further categorized by type, inheritance pattern, and segregation status when available. Population-frequency annotation (presence/absence in gnomAD [12]) and “novel variant” status were recorded when provided.

### 2.4. Data Collection and Phenotyping

Demographic and clinical data were extracted from medical records and genetic reports. Recorded variables included referral source, referral indication, and primary clinical reason for genetic evaluation. Phenotypic features were systematically coded across seven domains (growth, neurodevelopment, skin/pigmentation, skeletal/limb, organ malformations, immune/infectious, and hematologic), with facial dysmorphism recorded separately. Phenotypic burden was summarized as the number of positive domains and additionally summarized as a binary variable (≥2 domains vs. <2 domains) to distinguish patients with broader multisystem involvement from those with more limited phenotypic findings.

### 2.5. Patient Classification

To reflect the clinical spectrum of CPS referrals, patients were assigned to one of three mutually exclusive groups using a predefined hierarchical rule (A > B > C):

• Group A (Tumor/Malignancy): documented solid or hematologic malignancy/tumor.
• Group B (High-risk hematologic/immune phenotype without tumor): no documented tumor, but clinically significant hematologic and/or immune manifestations suspicious for inherited predisposition (e.g., persistent cytopenias, suspected inherited bone marrow failure phenotype, or immune dysregulation).
• Group C (Phenotype-only): neither tumor nor a high-risk hematologic/immune phenotype; referrals were driven predominantly by syndromic features (e.g., dysmorphism/congenital anomalies, neurodevelopmental delay/intellectual disability, growth abnormalities), suspected CPS, or familial/cascade testing in first-degree relatives. This group also included unaffected at-risk relatives undergoing cascade testing who were found to carry the familial pathogenic/likely pathogenic variant (“unaffected carriers”).

When overlapping features were present, classification followed the hierarchy A > B > C. Given the small size of Group B, inferential analyses were prespecified as a two-group comparison between high-risk presentation (Groups A + B combined) and phenotype-only (Group C), while three-group results were presented descriptively.

### 2.6. Statistical Analysis

Analyses were primarily descriptive. Categorical variables are presented as counts and percentages, and continuous variables as median and interquartile range (IQR). Comparisons between high-risk presentation (A + B) and phenotype-only (C) were performed using Fisher’s exact test for 2 × 2 categorical comparisons and chi-square tests for multi-category comparisons, as appropriate. For analyses involving multiple binary phenotypic domains, *p*-values were adjusted using the Holm method. A two-sided *p*-value < 0.05 was considered statistically significant. All numbers apart from *p*-values were rounded to one decimal place.

## 3. Results

Between 2020 and 2025, our unit provided genetic counseling and performed molecular genetic testing for 430 pediatric patients referred for a broad range of clinical indications. Of these, 42 patients met the predefined criteria for CPS and were included in the analytic cohort.

The cohort was predominantly male (71.4%) with a wide age distribution (10 days to 16 years), reflecting the heterogeneity of referral indications (Table 1). Using a predefined hierarchical classification, most patients were assigned to the phenotype-only group (Group C, 69.0%), while a smaller proportion presented with malignancy (Group A, 21.4%) or high-risk hematologic/immune features (Group B, 9.5%) (Table 1).

For inferential analyses, Groups A and B were prespecified as a combined high-risk presentation group (A + B) and compared against Group C.

### 3.1. Referral Pathways

**Figure 1:**
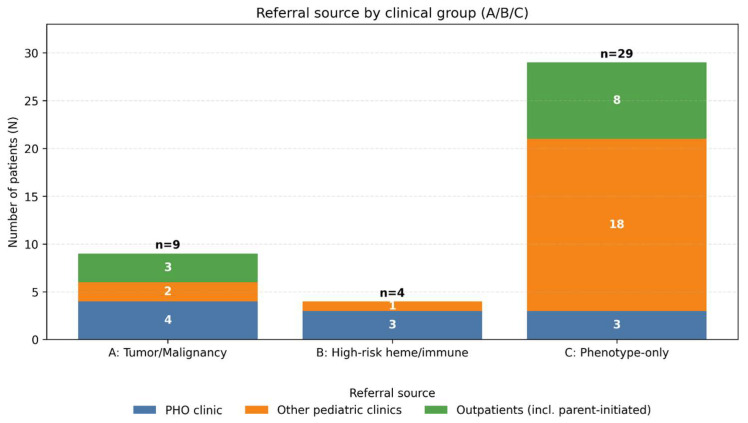
Referral source by clinical group (A/B/C).

Overall, most referrals originated outside the pediatric hematology-oncology (PHO) clinic of the hospital. Specifically, referrals came from other pediatric clinics (21/42, 50.0%) and outpatients, including parent-initiated evaluations (11/42, 26.2%), while 10/42 (23.8%) were referred via the PHO clinic (Figure 1; Table 1). PHO referrals were enriched in Group B (3/4, 75.0%) and Group A (4/9, 44.4%), whereas Group C was predominantly referred from other pediatric clinics (18/29, 62.1%) and outpatients (8/29, 27.6%), with only 3/29 (10.3%) referred via the PHO clinic. Overall, these findings demonstrate that most patients with suspected CPS are identified outside oncology services, with a substantial contribution from general pediatric practice. This highlights the key role of non-oncology clinicians in the early recognition and referral of patients with potential cancer predisposition.

### 3.2. Referral Indication, CPS Suspicion at Referral, and Primary Referral Reason

The recorded referral indication was most commonly syndrome-specific referral (23/42, 54.8%) or a non-specific clinical presentation (12/42, 28.6%), while familial/cascade testing accounted for 3/42 (7.1%) and non-specific CPS suspicion (SA) for 4/42 (9.5%) (Table 1). CPS was suspected at the time of referral in 29/42 (69.0%) cases, including all Group A patients (9/9, 100.0%), 3/4 (75.0%) in Group B, and 17/29 (58.6%) in Group C (Table 1).

**Figure 2:**
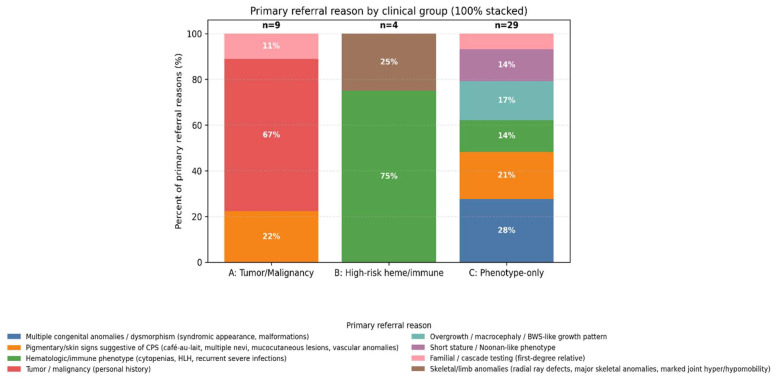
Primary referral reason by clinical group (100% stacked).

The most common primary referral reasons (i.e., main clinical drivers prompting genetic evaluation) were pigmentary/skin signs suggestive of CPS (8/42, 19.0%), multiple congenital anomalies/dysmorphism (8/42, 19.0%), hematologic/immune phenotypes (7/42, 16.7%), and tumor/malignancy (6/42, 14.3%). Growth-related referrals were also common: overgrowth/macrocephaly/BWS-like pattern (5/42, 11.9%) and short stature/Noonan-like phenotype (4/42, 9.5%) (Figure 2, Table S1). Tumor-driven referrals clustered in Group A (6/9, 66.7%), whereas hematologic/immune referrals clustered in Group B (3/4, 75.0%); referrals prompted by multiple congenital anomalies/dysmorphism were most frequent in Group C (8/29, 27.6%) (Table S1).

These results indicate that syndromic and non-specific clinical features are major drivers of referral for genetic evaluation, even in the absence of malignancy. This underscores the importance of careful clinical assessment in identifying patients at risk for CPS.

To provide a concise overview of key clinical and molecular characteristics by analysis group, we summarize selected features in Table 2, followed by detailed phenotypic and genetic results below.

### 3.3. Phenotypic Profile and Burden

Phenotypic features were common across the cohort and varied across clinical groups. Facial dysmorphism was recorded in 25/42 (59.5%), most frequently in Group C (21/29, 72.4%) (Table S2). Overall, 29/42 (69.0%) patients had involvement of ≥2 phenotypic domains, including 4/4 (100.0%) in Group B and 22/29 (75.9%) in Group C (Table S2). At the domain level, skin/pigmentation findings were present in 9/42 (21.4%), whereas hematologic precursor features were present in 7/42 (16.7%) and were particularly frequent in Group B (3/4, 75.0%) (Table S2).

**Figure 3:**
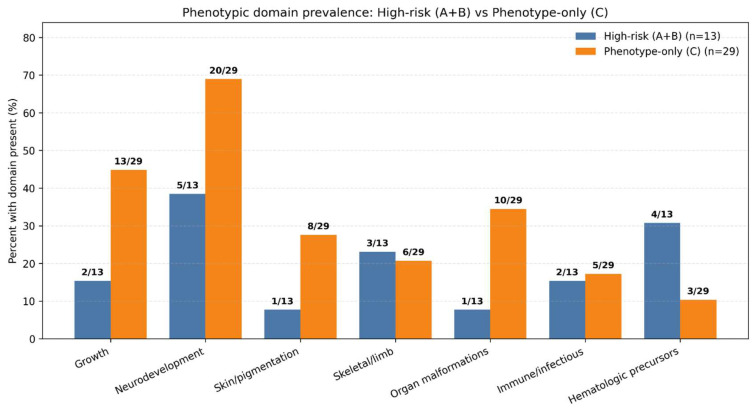
Phenotypic domain prevalence in high-risk presentation (A + B) versus phenotype-only (C).

No statistically significant differences were observed between the high-risk and phenotype-only groups across the analyzed clinical domains after adjustment for multiple comparisons. However, trends toward a higher prevalence of growth abnormalities, neurodevelopmental features, and organ malformations were noted in the phenotype-only group, while hematologic precursor conditions appeared more frequent in the high-risk group (Figure 3; Table S2).

The high prevalence of multisystem involvement, particularly in the phenotype-only group, highlights the heterogeneous and often non-oncologic presentation of CPS. These findings support the importance of comprehensive phenotypic evaluation in guiding genetic testing and diagnosis.

### 3.4. Genetic Testing and Molecular Findings

Genetic testing was performed externally and most commonly involved targeted multigene panel testing (24/42, 57.1%) and WES/clinical exome sequencing (15/42, 35.7%), while imprinting/methylation testing and other targeted approaches were uncommon (3/42, 7.1%) (Table S3). Inheritance was predominantly autosomal dominant (34/42, 81.0%), followed by autosomal recessive (6/42, 14.3%) and other/unknown inheritance (2/42, 4.8%) (Tables S3 and S4).

At the patient level, 38/42 (90.5%) had at least one pathogenic/likely pathogenic (P/LP) variant, 2/42 (4.8%) had VUS-only findings, and 2/42 (4.8%) had diagnostic variants reported without an explicitly stated ACMG class; variant-level classifications (when provided) comprised 32 P, 8 LP, and 3 VUS, reflecting that some autosomal recessive diagnoses involved two reported variants (Tables S3 and S5). VUS findings were considered clinically contributory when phenotype–gene concordance was strong, while retaining the laboratory classification as VUS (Table S5).

Variant classes were primarily loss-of-function/truncating (18/42, 42.9%) and missense (17/42, 40.5%), with less frequent splice-site variants (3/42, 7.1%) and CNVs (2/42, 4.8%) (Table S3). Among autosomal dominant inherited cases, an affected parent was documented in 14/34 (41.2%) (Tables S3 and S5). When population-frequency annotation was available, 25/40 (62.5%) variants were absent from gnomAD [12], and 9/38 (23.7%) were reported as previously unreported in public databases at the time of reporting (Tables S3 and S5).

**Figure 4:**
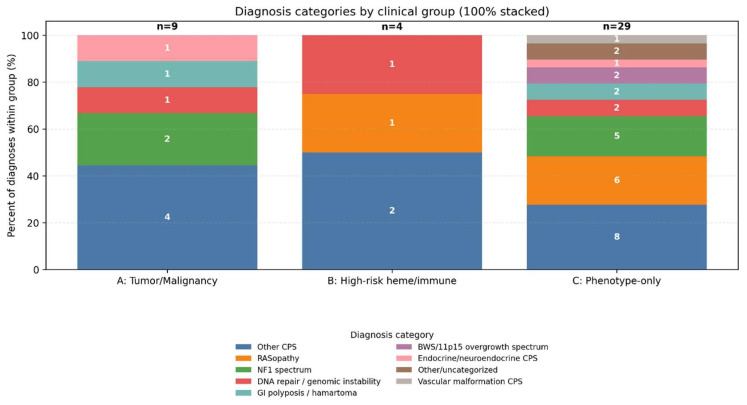
Diagnosis categories in high-risk presentation (A + B) versus phenotype-only (C) (100% stacked).

To provide a clinically interpretable overview of the molecular diagnoses, conditions were grouped into diagnosis categories (Table S6). The most frequent categories were NF1 spectrum (8/42, 19.0%) and RASopathies (7/42, 16.7%), followed by DNA repair/genomic instability disorders (4/42, 9.5%), GI polyposis/hamartoma syndromes (3/42, 7.1%), and BWS/11p15 overgrowth spectrum (2/42, 4.8%) (Table S6). In the prespecified A + B versus C comparison, the overall distribution of diagnosis categories did not differ significantly (χ^2^ test, *p* = 0.744; sparse cells), and group-specific patterns are shown in Figure 4 (Table S6; Figure 4).

The high rate of pathogenic or likely pathogenic findings reflects the selected nature of the cohort and the effectiveness of current genetic testing approaches. These results further emphasize the central role of genetic diagnostics in confirming CPS and guiding clinical management.

Detailed case-level molecular and phenotypic information for all 42 patients is provided in Supplementary Table S5.

Overall, our findings highlight three key aspects of genetic counseling for CPS in pediatric practice: a substantial proportion of patients are identified outside oncology settings; multisystem phenotypic features remain a major driver of referral, and genetic testing yields a high rate of clinically relevant findings, predominantly involving autosomal dominant conditions.

## 4. Discussion

CPS are being identified with increasing frequency in pediatric oncology. This trend likely reflects not only a true underlying burden but also the growing use of molecular genetic testing in clinical practice, which has substantially enhanced the detection of germline alterations [13].

These findings raise an important question: who should be referred for genetic counseling, and by whom? Traditionally, children with cancer were referred for genetic evaluation when clinicians identified a personal and/or family history suggestive of a CPS [4]. While family history remains an important clinical tool, it is absent in approximately 40–60% of pediatric patients with CPS [4], limiting its utility as a primary referral criterion. Consequently, reliance on a positive family history alone is no longer sufficient for identifying at-risk individuals. Notably, our results show that the majority of patients in this cohort were referred by general pediatricians based on non-oncologic features of an underlying CPS, rather than on the presence of malignancy itself. This finding underscores the critical role of non-oncology pediatricians in the early recognition of CPS and highlights the need for increased awareness of syndromic features suggestive of cancer predisposition in routine pediatric practice.

Although differences in phenotypic domain distribution between high-risk and phenotype-only groups did not reach statistical significance after correction for multiple comparisons, several clinically relevant trends were observed. Neurodevelopmental abnormalities, growth disturbances, and organ malformations were more frequent in the phenotype-only group, whereas hematologic precursor conditions were more common among high-risk patients. This fact reflects the heterogeneous and often non-oncologic presentation of these syndromes, particularly in early childhood, when malignancy may not yet have developed. For example, optic pathway gliomas in Neurofibromatosis type 1 typically arise in early childhood, often before the age of seven [14], whereas characteristic features such as multiple café-au-lait macules may be present from birth and can serve as early clinical indicators of the condition [15]. Thus, in syndromic cancer predisposition, non-malignant features frequently precede the development of tumors and may provide the first opportunity for recognition of the underlying disorder.

Within this diagnosis-confirmed CPS cohort, most patients had clinically actionable germline findings reported in established CPS genes, predominantly involving autosomal dominant conditions. This finding is consistent with previous studies demonstrating that germline alterations in tumor suppressor genes, frequently inherited in an autosomal dominant manner, represent a major proportion of CPS [16].

The most frequently affected gene in our cohort was NF1, which aligns with this pattern. Given that the prevalence of Neurofibromatosis type 1 is estimated at approximately 1:2000–3000 individuals [14], its predominance within our cohort is not surprising. Notably, an affected parent was identified in 14/34 (41.2%) cases with available inheritance data. This finding raises important considerations regarding the organization and accessibility of genetic counseling services in our country. In many instances, parents were unaware that they themselves carried a genetic condition associated with an increased cancer risk and the potential for transmission to offspring. As a result, opportunities for early identification, surveillance, and reproductive counseling, including prenatal or preimplantation genetic testing, may have been missed [17]. Moreover, familial/cascade testing accounted for only 3/42 (7.1%) of cases, representing a relatively low proportion and suggesting potential gaps in the organization and implementation of genetic services in our country.

Nevertheless, our findings support the growing role of genetic counseling as an integral component of pediatric oncology care. Beyond facilitating diagnosis, genetic counseling contributes to risk stratification, surveillance planning, and family counseling, including cascade testing in at-risk relatives [4]. The identification of unaffected carriers within our cohort further underscores the preventive potential of such services, allowing for early monitoring and intervention before the onset of malignancy.

This study has several limitations. Its retrospective design and relatively small sample size limit the statistical power to detect significant differences between subgroups. Additionally, the analytic cohort represents a selected, diagnosis-confirmed CPS subset, and therefore proportions reported in this manuscript describe distributions within this cohort and should not be interpreted as population prevalence or diagnostic yield among all referrals. Referral patterns reflect local healthcare structures and may not generalize directly to other settings. Finally, long-term clinical outcomes and the impact of genetic findings on patient management were not systematically assessed due to the lack of universal electronic health records in our country.

Despite these limitations, our study provides valuable insight into the real-world implementation of genetic counseling for CPS in a regional tertiary care setting. It highlights the importance of multidisciplinary collaboration, the continued relevance of clinical phenotyping, and the expanding role of genomic diagnostics in pediatric oncology and beyond.

## 5. Conclusions

Genetic counseling for childhood CPS is increasingly relevant across a broad spectrum of pediatric practice. Our experience demonstrates that many at-risk patients are identified outside oncology settings, often based on non-oncologic and syndromic features rather than malignancy itself. Increasing awareness of syndromic features, improving referral pathways, and expanding access to genetic services are essential steps toward optimizing the diagnosis, management, and prevention of hereditary cancer in children.

## Figures and Tables

**Table 1:** Baseline characteristics and referral context of the CPS cohort by clinical group (A/B/C).

Characteristic	Total (n = 42)	Group A (n = 9)	Group B (n = 4)	Group C (n = 29)
Age at evaluation, years, median (IQR)	5.0 (2.0–9.0) [n = 33]	4.5 (1.8–6.5) [n = 8]	8.5 (4.8–12.2) [n = 2]	6.0 (2.0–9.5) [n = 23]
Male sex, n (%)	30 (71.4%)	6 (66.7%)	2 (50.0%)	22 (75.9%)
Female sex, n (%)	12 (28.6%)	3 (33.3%)	2 (50.0%)	7 (24.1%)
Referral source: Pediatric oncology, n (%)	10 (23.8%)	4 (44.4%)	3 (75.0%)	3 (10.3%)
Referral source: Other pediatric clinics, n (%)	21 (50.0%)	2 (22.2%)	1 (25.0%)	18 (62.1%)
Referral source: Outpatients (incl. parent-initiated), n (%)	11 (26.2%)	3 (33.3%)	0 (0.0%)	8 (27.6%)
Personal history of tumor/malignancy, n (%)	9 (21.4%)	9 (100.0%)	0 (0.0%)	0 (0.0%)
Referral indication: Syndrome-specific, n (%)	23 (54.8%)	5 (55.6%)	3 (75.0%)	15 (51.7%)
Referral indication: Unclear phenotype, n (%)	12 (28.6%)	0 (0.0%)	1 (25.0%)	11 (37.9%)
Referral indication: Familial/cascade testing, n (%)	3 (7.1%)	1 (11.1%)	0 (0.0%)	2 (6.9%)
Referral indication: Non-specific CPS suspicion (SA), n (%)	4 (9.5%)	3 (33.3%)	0 (0.0%)	1 (3.4%)
CPS suspected at referral, n (%)	29 (69.0%)	9 (100.0%)	3 (75.0%)	17 (58.6%)

**Table 2:** Key phenotypic and selected molecular characteristics of the CPS cohort by analysis group (High-risk A + B vs. Phenotype-only C).

Characteristic	Total (n = 42)	High-Risk (A + B) (n = 13)	Phenotype-Only (C) (n = 29)	*p*-Value
Facial dysmorphism	25/42 (59.5%)	4/13 (30.8%)	21/29 (72.4%)	0.018
≥2 phenotypic domains	29/42 (69.0%)	7/13 (53.8%)	22/29 (75.9%)	0.173
Growth domain	15/42 (35.7%)	2/13 (15.4%)	13/29 (44.8%)	0.089
Short stature	10/42 (23.8%)	2/13 (15.4%)	8/29 (27.6%)	0.466
Tall stature/overgrowth	4/42 (9.5%)	0/13 (0.0%)	4/29 (13.8%)	0.293
Neurodevelopment domain	25/42 (59.5%)	5/13 (38.5%)	20/29 (69.0%)	0.092
Skin/pigmentation domain	9/42 (21.4%)	1/13 (7.7%)	8/29 (27.6%)	0.232
Skeletal/limb domain	9/42 (21.4%)	3/13 (23.1%)	6/29 (20.7%)	1
Organ malformations domain	11/42 (26.2%)	1/13 (7.7%)	10/29 (34.5%)	0.127
Immune/infectious domain	7/42 (16.7%)	2/13 (15.4%)	5/29 (17.2%)	1
Hematologic precursors domain	7/42 (16.7%)	4/13 (30.8%)	3/29 (10.3%)	0.176
Autosomal dominant inheritance (AD)	34/42 (81.0%)	11/13 (84.6%)	23/29 (79.3%)	1
Autosomal recessive inheritance (AR)	6/42 (14.3%)	2/13 (15.4%)	4/29 (13.8%)	1
LoF/truncating variant class	18/42 (42.9%)	9/13 (69.2%)	9/29 (31.0%)	0.041
Missense variant class	17/42 (40.5%)	4/13 (30.8%)	13/29 (44.8%)	0.505
Affected parent documented	14/42 (33.3%)	5/13 (38.5%)	9/29 (31.0%)	0.729
gnomAD absent	28/42 (66.7%)	7/13 (53.8%)	21/29 (72.4%)	0.298

## Data Availability

The original contributions presented in this study are included in the article/Supplementary Materials. Further inquiries can be directed to the corresponding author.

## Supplementary Materials

The following supporting information can be downloaded at: https://www.mdpi.com/article/10.3390/children13060793/s1, Table S1: Primary reason for testing of the patients, included in the studied domains; Table S2: Characteristics of the studied domains; Table S3: Summary of the genetic findings; Table S4: List of the reported genes; Table S5: Genetic results of the included participants; Table S6: Studied diagnostic categories—overall and by groups.

## Abbreviations

The following abbreviations are used in this manuscript:

ACMG/AMP	American College of Medical Genetics and Genomics/Association for Molecular Pathology
BWS	Beckwith–Wiedemann syndrome
CNV	Copy number variation
CPS	Cancer predisposition syndromes
gnomAD	Genome Aggregation Database
IQR	Interquartile range
LP	Likely pathogenic
NF1	Neurofibromatosis type 1
NGS	Next-generation sequencing
PHO	Pediatric hematology-oncology
P/LP	Pathogenic/Likely pathogenic
VUS	Variant of uncertain significance
WES	Whole exome sequencing
